# Human Rhinoviruses in Pediatric Patients in a Tertiary Care Hospital in Germany: Molecular Epidemiology and Clinical Significance

**DOI:** 10.3390/v14081829

**Published:** 2022-08-20

**Authors:** Franziska Neugebauer, Sandra Bergs, Uwe Gerd Liebert, Mario Hönemann

**Affiliations:** Virology Department, Institute of Medical Microbiology and Virology, Leipzig University, Johannisallee 30, 04103 Leipzig, Germany

**Keywords:** rhinovirus, molecular epidemiology, respiratory infections, respiratory viruses, pediatric patients

## Abstract

Rhinoviruses (RVs) constitute a substantial public health burden. To evaluate their abundance and genetic diversity in pediatric patients, RV RNA in respiratory samples was assessed using real-time RT-PCR and partial nucleic acid sequencing of viral genomes. Additionally, clinical data were retrieved from patient charts to determine the clinical significance of pediatric RV infections. In total, the respiratory specimens of 776 patients (<18 years), collected from 2013 to 2017, were analyzed. Infections occurred throughout the entire year, with peaks occurring in fall and winter, and showed remarkably high intra- and interseasonal diversity for RV genotypes. RV species were detected in the following frequencies: 49.1% RV-A, 5.9% RV-B, and 43.6% RV-C. RV-C was found to be more frequently associated with asthma (*p* = 0.04) and bronchiolitis (*p* < 0.001), while RV-A was more frequently associated with fever (*p* = 0.001) and pneumonia (*p* = 0.002). Additionally, 35.3% of the patients had co-infections with other pathogens, which were associated with a longer hospital stay (*p* < 0.001), need for ventilation (*p* < 0.001), and pneumonia (*p* < 0.001). Taken together, this study shows pronounced RV genetic diversity in pediatric patients and indicates differences in RV-associated pathologies, as well as an important role for co-infections.

## 1. Introduction

Rhinoviruses (RVs) are highly prevalent and can cause both upper and lower respiratory tract infections (URTIs and LRTIs). The clinical spectrum includes common colds [1], otitis media, and sinusitis [2,3,4,5], as well as more severe conditions, such as bronchiolitis and pneumonia [2,6,7]. Moreover, RVs have been associated with the development and exacerbation of asthma and wheezing in children (allergic sensitization) [8,9] and are the main viral cause of exacerbations of chronic obstructive pulmonary disease (COPD) in adults [10,11,12,13]. Rhinoviruses (RVs) belong to the genus *Enterovirus* (EV) within the family of *Picornaviridae*. At least 169 different RV genotypes are known, which are subdivided into three species: RV-A, RV-B, and RV-C [14]. While RV-A and RV-B have been known since the 1950s, RV-C was only first identified in 2006, as it cannot be propagated in standard cell cultures [15,16,17]. RVs are non-enveloped RNA viruses with a positive-sense, single-stranded genome comprising approximately 7200 nucleotides and encoding both viral structural and non-structural proteins (VPs and NSPs) [18]. Four proteins, named VP1, VP2, VP3, and VP4, form the viral capsid that encases the genome and interacts with the cell surface receptors. For most RV-A and RV-B serotypes (“major group”), the intercellular adhesion molecule 1 (ICAM-1) represents the cellular receptor, while a minority (“minor group”) utilize the low-density lipoprotein receptor (LDLR) [2]. Cadherin-related family member 3 (CDHR3) is the cellular receptor for RV-C [19].

Unlike human enteroviruses or other members of the *Picornaviridae* family, such as parechoviruses, the lack of capsid recombination is a characteristic of RV [20]. Thus, the genomic regions of VP1 and VP4/VP2 were both proposed for genotypic classification. While the VP4/VP2 regions allow the genotypic analysis of all three RV species with the same protocol, the VP1 region offers the highest precision and represents the cornerstone for the assignment of newly discovered genotypes [21].

Though they represent an important public health burden, RVs’ epidemiology and significance remain insufficiently studied. To evaluate the abundance of RVs and the local genetic diversity of RVs in pediatric patients, the molecular epidemiology of RV was assessed and analyzed between 2013 and 2017 at a tertiary care hospital in Germany. Additionally, the species-specific clinical significance of the RV cases was analyzed based on the clinical data for the same patient group.

## 2. Materials and Methods

### 2.1. Sample Collection and Clinical Data 

From 2013 to 2017, 5251 respiratory samples from 3533 pediatric in- and outpatients (<18 years) were collected and tested for viral respiratory infections. Samples included nasal and/or naso-oropharyngeal swabs (84.5%, n = 4435), nasal secretions (7.3%, n = 385), sputum (0.2%, n = 8), throat rinsing fluid (1.5%, n = 80), tracheal secretions (5%, n = 260), and broncho-alveolar lavage fluids (1.6%, n = 83). Testing was initiated at the discretion of the attending physician. To avoid a bias caused by follow-up samples, re-testing within six weeks after the initial detection was defined as a single case. Data relating to underlying medical conditions and clinical parameters on the day of RV detection were retrieved retrospectively from patient charts. In the case of missing clinical information, (n/total) indicates the respective cases for the total amount of available data. The body temperature was categorized as follows: normal (<37.5 °C), subfebrile (37.5–37.9 °C), and fever (≥38.0 °C). The classification of URTIs and LRTIs was carried out according to the International Statistical Classification of Diseases and Related Health Problems (ICD-10- WHO) and the diagnoses and information listed in the patients’ records. Bacterial and fungal pathogens were cultivated with standard microbiological techniques and considered as co-infections if a detection from respiratory samples or blood was documented. Viral co-infections were assessed through a multiplex test for respiratory viruses (see below) and through established routine laboratory protocols for CMV [22,23], EBV [24], HHV-6 [25], and HSV [26]. RV seasons were defined as starting on 1st October and ending on 30th September of the following year. The Leipzig University Ethics committee approved the study design (no. Az 301/16-ek).

### 2.2. Nucleic Acid (NA) Extraction and RV Detection 

Total NA was extracted from 200 μL of each respiratory sample using the DNA and Viral NA Small Volume Kit with a MagNA Pure 96 instrument (both, Roche, Mannheim, Germany) according to the manufacturer’s instructions. Nucleic acids were stored in aliquots at −80 °C until further use. The presence of genomes of common respiratory viruses was assessed using a multiplex panel assay (NxTAG RPP, Luminex corporation, Austin, TX, USA) according to the manufacturer’s instructions. The panel included influenza viruses A and B, respiratory syncytial viruses A and B, parainfluenza viruses 1 to 4, human coronaviruses (including 229E, NL63, OC43, and HKU1), human metapneumoviruses, adenoviruses, human bocaviruses, rhinoviruses, and enteroviruses. Samples that reacted to the combined enterovirus/rhinovirus target of the assay were further analyzed to determine the presence of rhinovirus-specific RNA. In brief, one-step real-time RT-PCR was performed using the QuantiFast^®^ Multiplex kit (Qiagen, Hilden, Germany) based on a previously proposed protocol and a modified forward primer, which provides increased binding strength due to the incorporation of locked nucleic acids (5’-CY+AGCCTGCGTGGC-3′) [27]. For a detailed description of the reaction conditions, see Supplementary Tables S1–S3.

### 2.3. Rhinovirus Genotyping and Phylogenetic Analysis 

Partial viral VP1 and VP4/VP2 genes were amplified for viral genotyping [28,29] using the BigDye Terminator Sequencing Kit v1.1 and an ABI 3500 Genetic Analyzer (both Applied Biosystems, Foster City, CA, USA). The amplicon sizes depended on the RV genotype and were about 340 bp for VP1 and 420 bp for VP4/VP2. If multiple RV-RNA-positive samples from the same patient were available, the first one was used for genotyping. The obtained sequences were submitted to GenBank (accession numbers ON767112 to ON768616). Separate phylogenetic trees for VP1 and VP4/VP2 were constructed at the nucleotide level using MEGA software, version 6, based on the maximum likelihood method. Bootstrap analysis was performed with 1000 replicates [30]. The phylogenetic trees included reference sequences proposed by the Picornavirus Study Group [14].

### 2.4. Statistical Analysis 

Statistical analyses were performed using IBM SPSS Statistics for Windows, version 24.0 (IBM Corp., Armonk, NY, USA). Continuous values were expressed as means or medians (range) and categorical data as frequencies (percentages). A Mann–Whitney U test or a Kruskal–Wallis test was performed to compare continuous variables. A Chi-square test was performed for categorical variables. Brackets indicate parameters that were analyzed in the same contingency table. All tests were two-tailed. A *p*-level of <0.05 was considered significant. For subsequent pairwise comparisons of column proportions, a Bonferroni correction for multiple comparisons was applied.

## 3. Results

### 3.1. Species Distribution and Seasonality

**Figure 1:**
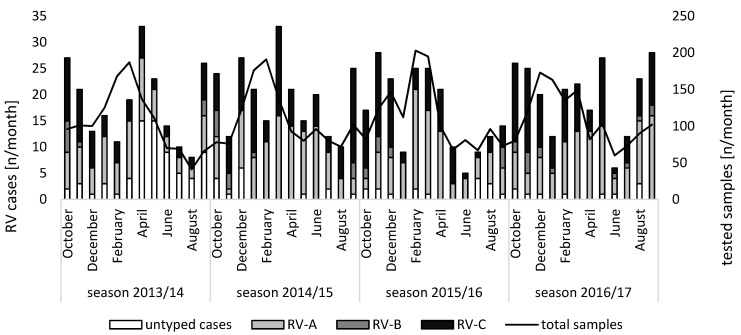
Monthly total numbers of tested samples and rhinovirus cases (n = 776) stratified by species A, B, and C, as well as untypable rhinoviruses. Note the two different y-axes: the left axis shows the absolute numbers of detected rhinovirus A, rhinovirus B, and rhinovirus C cases, as well as the absolute numbers of untyped cases, while the right axis shows the absolute number of tested samples.

In total, the presence of RV RNA was confirmed in 969 samples (18.5%) by RT-qPCR. Positivity rates ranged from 17.3% (233/1347) in the season of 2015/2016 to 19.4% (246/1271) in the season of 2013/2014, while they were 18% (239/1330) in the season of 2016/2017 and 19.2% (250/1303) in the season of 2014/2015. Altogether, 884 cases were identified. RV infections occurred throughout the whole year without strict seasonality. However, an increased prevalence was observed between September and April. Additionally, a dip in January was noted for RV detections in all seasons (Figure 1). The combined cases per month are presented in Supplementary Figure S1. Partial gene amplification was successful for 776 (87.8%) cases. Of those, 750 (96.6%) cases could be genotyped in the VP4/VP2 region and 749 (96.5%) cases in the VP1 region. The genotype was assigned with only the partial VP4/VP2 or VP1 gene region for 26 (3.4%) or 27 (3.5%) cases, respectively. The genotyping approach was unsuccessful for both genetic regions in 108 cases (12.2%). The cases with a successful genotypic analysis were analyzed further. Within the genotyped subset, respiratory specimens included 646 nasal and/or oropharyngeal swabs (83.2%), 69 nasal secretions (8.9%), 41 tracheal secretions (5.3%), 13 throat rinsing fluids (1.7%), and 7 broncho-alveolar lavage fluids (0.9%). Of these, 148 samples (19.1%) were collected in 2013/2014, 219 (28.2%) in 2014/2015, 180 (23.2%) in 2015/2016, and 229 (29.5%) in 2016/2017 (Table 1). Overall, RV-A predominated with 381 detections (49.1%), followed by RV-C with 338 (43.6%) and RV-B with 46 (5.9%) detections. While RV-A and RV-C could be found throughout the entire year, RV-B was not detected from February to May in any of the seasons. An alternating predominance of RV-A and RV-C was observed between April 2014 and December 2016.

**Figure 2:**
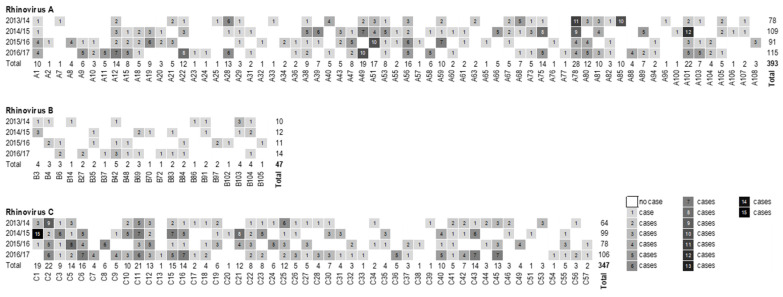
Heat map of the total numbers of RV genotypes (n = 787) detected during the study period stratified by season.

Overall, 80.5% of the established RV genotypes (136/169) were detected in the present case cohort. Of these, 17 genotypes were identified in all four seasons—RV-A1, RV-A12, RV-A38, RV-A53, RV-A56, RV-A78, RV-A101, RV-C2, RV-C3, RV-C5, RV-C11, RV-C12, RV-C15, RV-C16, RV-C22, RV-C25, and RV-C41—and another 34 could be detected in three seasons (Figure 2). Regarding the whole study period, the most frequent genotypes of each species were: RV-A78 (3.6%, n = 28/787), RV-C2 (2.8%, n = 22/787), and RV-B42 (0.6%, n = 5/787). The genotype that was most abundant in a single season was RV-C1, with 15 detections in the season of 2014/2015 (6.8%, n = 15/220). When a genotype was detected in a respective season, the average rate was 2.8 times for RV-A and RV-C, while it was 1.3 times for RV-B. No seasonal pattern was observed for the occurrence of specific genotypes, either within or between the studied seasons. In 11 cases (1.4%), a co-infection with two different RV genotypes was found. Six co-infections with different RV species (RV-A59/RV-C15, RV-A20/RV-C41, RV-A20/RV-C32, RV-A80/RV-C4, RV-A51/RV-C32 and, RV-A67/RV-B42) and five with the same RV species (RV-A19/RV-A38, RV-A12/RV-A78, RV-A9/RV-A15, RV-C1/RV-C41 and, RV-C11/RV-C56) were noted.

The RV co-infections with the same RV species were identified through a divergent genotyping result in both analyzed partial gene regions. The RV co-infections with different RV species were identified through genotypic analysis of the partial VP1 gene region subsequent to either inconclusive results (n = 3) or a minor variant (n = 3) in the analysis of the partial VP4/VP2 gene region. For the other 23 samples for which VP4/VP2 sequencing was unsuccessful, the corresponding VP1 genotypes were RV-A11, RV-A19, RV-A28, RV-A31, RV-A38, RV-A51, RV-A53, RV-A56, RV-A58, RV-A73, RV-A75, RV-A81, RV-A101, RV-B91, RV-B103 (n = 2), RV-B104, RV-C11 (n = 3), RV-C40, and RV-C43 (n = 2). For the 27 samples for which VP1 sequencing was unsuccessful, the corresponding VP4/VP2 genotypes were RV-A31, RV-A56, RV-C2, RV-C5, RV-C7 (n = 2), RV-C8, RV-C9 (all cases, n = 5), RV-C11, RV-C19, RV-C21 (n = 2), RV-C25 (n = 2), RV-C26, RV-C28 (n = 2), RV-C30, RV-C39 (all cases, n = 1), RV-C49, RV-C55, and RV-C56 (all cases, n = 2). In the remaining subset of cases with both VP1 and VP4/VP2 genes (92.1%, n = 715/776), the genotyping results for the two genetic regions matched.

### 3.2. Study Population and Clinical Features 

The mean age of the study population was 2.4 years (median age 1 year), which did not differ significantly between RV species or between seasons. With regard to gender, 41% of all patients were female and 59% were male. Again, no differences were noted between seasons or between RV species (Table 1 and Table 2). 

The RV species-specific patient characteristics and clinical parameters are presented in Table 2. Cases with detections of two different RV genotypes were excluded from the analysis (n = 11). 

In the pairwise analysis, significant differences were mainly found between RV species A and C. The length of stay in hospital was higher for RV-A (*p* = 0.007). Asthma was observed more frequently in the RV-C cases (*p* = 0.009), while cardiovascular preconditions were more frequently associated with RV-A (*p* = 0.004). RV-A was associated with a higher frequency of fever (*p* = 0.009), while RV-C was associated with a higher amount of cases with normal temperature (*p* < 0.001). No differences were noted for temperatures between 37.5 and 37.9 °C. RV-C was associated with a higher amount of cases that presented with dyspnea (*p* = 0.007), bronchodilator usage (*p* < 0.001), and bronchiolitis (*p* < 0.001), while RV-A was associated with more pneumonia cases (*p* = 0.001). The highest number of co-infections with other pathogens was observed for RV-A (*p* < 0.001), while the composition of the pathogen groups did not differ between the three RV species. For RV-B, dyspnea (*p* = 0.011), bronchodilator usage (*p* = 0.017), and bronchiolitis (*p* = 0.017) were found less frequently when compared to RV-C. No statistical differences were found for comparisons between RV species B and A.

### 3.3. Co-Infections 

Cases with detections of two different RV genotypes were excluded from the analysis (n = 11). In total, 270 (35.3%) cases with co-infections were identified, of which 99 (12.9%) were bacterial, 143 (18.7%) were viral, and 1 (0.1%) was fungal. The bacterial co-infections included 30 cases with detections of more than one bacterial species. The viral co-infections included 26 cases with detections of more than one additional viral pathogen. In 27 cases (3.5%), co-infections with at least two different pathogen types were observed. The most frequently detected bacterial pathogens were *Haemophilus influenzae* (n = 26), *Staphylococcus aureus* (n = 19), and *Streptococcus pneumoniae* (n = 17). Regarding viral co-pathogens, adenovirus (n = 61), bocavirus (n = 44), and respiratory syncytial virus (n = 44) were found most frequently (Table 3). 

The comparison of RV infections with and without co-infections is presented in Table 4. RV-A was more frequently detected in cases with a co-infection (*p* < 0.001) than RV-C, while RV-C was more frequently associated with an infection without the detection of other pathogens (*p* < 0.001). Co-infections were associated with a prolonged length of stay, a higher frequency of symptomatic infections, and fever (*p* = 0.013). Furthermore, a higher number of cases with co-infections showed pharyngitis, tonsillitis, and pneumonia, as well as the need for an ICU stay of any length and the need for invasive ventilation (*p* < 0.001). Infections with only RV detections were associated with a higher frequency of normal temperature (*p* = 0.008) and no need for ventilation (*p* < 0.001).

## 4. Discussion

The detection of identical genotypes all over the globe seemingly indicates a rapid spread of rhinoviruses without geographic restrictions [21]. Consistent with this observation, 80.5% of the currently proposed RV genotypes were found, despite a limited sample set and a local sampling approach. A global circulation pattern is therefore difficult to assess, as even a major migration wave, such as the one observed in Europe in the wake of the 2015 Syrian refugee crisis, seemingly had a minimal impact on the detected seasonal genotype structure. Despite displaying rapidly changing genotype compositions, with the most common genotype RV-A78 representing only 3.6% (28/787) of all detected rhinovirus genotypes, 37.5% of the detected genotypes (51/136) were observed in at least three of the four analyzed seasons. In the corresponding adult cohort of the same time period, 18% of the detected genotypes (20/111) were shown to circulate in at least three seasons [23]. Additionally, the positivity rate was lower (4.3%) in the adult cohort when compared to the pediatric cohort. A possible explanation might be a more prevalent and diverse RV circulation in children that is favored by a naïve immune system. The subsequent development of serotype-specific humoral immune responses, representing a correlate of an (at least) transient immunity [31], could therefore lead to a more restricted genotype profile and RV abundance later in life. Furthermore, the occurrence of locally restricted and time-limited rhinovirus outbreaks of distinct genotypes has been hypothesized before [32]. The average amount of genotype detections per season in the current study was 2.8 times for RV-A and RV-C. Thus, with an arbitrary cutoff for detection rates of more than three times the expected average, the detections of RV-A78 and RV-A85 in season 2013/14, RV-A101 and RV-C1 in 2014/15, RV-A51 in season 2015/16, and RV-A49 in season 2016/17 may represent localized outbreaks. The possibility of expanding the outbreak hypothesis to a third quality—namely, age—may further contribute to the subtle differences noted in the observed seasonal RV prevalence between the adult and pediatric cases. The most prevalent genotype RV-A78 was observed only three times in the adult cohort, and only once in season 2013/14 for this cohort. Conversely, 10 genotypes of species RV-A were detected more frequently than the most prevalent genotype of the adult cohort, RV-A1. In agreement with the current study, in a meta-analysis of 31 studies covering genotyping results of all three RV species [33], RV-A78, RV-A12, and RV-C2 were found to be the most abundant genotypes, representing 10% of all detections. The included studies covered a period of 13 years (2006 to 2019), which might be too narrow a timeframe for the observation of larger shifts in genotype circulation patterns. Of note, further genotypes were observed with a high frequency in the current study (RV-A49, RV-A56, RV-A75, RV-A101, RV-C1, RV-C5, RV-C6, RV-C11, RV-C15, RV-C16, and RV-C43), which may indicate regional shifts in genotype predominance. It is therefore arguable that larger population-based studies over an extended period of time are needed to increase the understanding of rhinovirus circulation.

In the present study, rhinovirus detections showed two pronounced peaks around September/October and April with varying dips, but detections trended in January and the summer months, which is in line with earlier reports [2,34]. However, the seasonality showed slight differences from what was seen in the corresponding adult cohort [23] and another European study from Amsterdam with a continuous drop after February throughout the spring months [35]. A marked alternating predominance of RV-A and RV-C was only observed for a part of the study period. The RV species distribution was previously reported with values of 44–75.9% for RV-A, 0–18.2% for RV-B, and 20–55% for RV-C [33]. Although the present study is in line with these observations, a substantial impact with regard to age and the number of co-infections in the investigated patient cohort needs to be considered. RV-C (43.6%) was detected nearly as often as RV-A (49.1%) (Table 1). If only considering the subset of RV infections with a co-infection of another pathogen, a substantial shift to rates of 59.3% for RV-A and 34.8% for RV-C can be observed. This species composition resembles the one that was reported for the corresponding adult cohort [23], which was 60.9% RV-A, 12.7% RV-B, and 26.4% RV-C.

The assessment of the clinical presentation of RV, and even species-specific pathology, is very complex and may be influenced by additional co-factors. Individual comorbidities, such as asthma or a CDHR3 polymorphism [36,37], have to be considered, as well as infections with other pathogens. Additionally, the high genetic diversity of RVs and divergent study designs with different study populations impose major challenges regarding data interpretation and comparability. 

Species-specific differences in clinical presentation and severity have been reported in some studies; however, the symptoms of individual patients do not seem to allow a differentiation between the three RV species. In addition to being more prevalent, RV-A and RV-C are increasingly associated with a higher clinical severity [33]. In the current study, RV-A was more frequently associated with fever and pneumonia, whereas RV-C was more frequently associated with bronchiolitis, dyspnea, and bronchodilator usage. In comparison, no differences in the clinical presentation were noted for the adult cohort of the same hospital [23]. In studies from Spain, China, and Vietnam, both RV-A and RV-C were found to be frequently associated with asthma, bronchiolitis, and pneumonia; however there were no statistical differences between the two species [38,39,40]. In a prospectively analyzed cohort of children with fever in Tanzania, no differences between any of the three RV species with regard to the clinical presentation could be deduced [41]. In a study from Taiwan, RV-A was more frequently associated with ICU admissions [42]. In line with the current study, pneumonia was observed more frequently in RV-A cases, while bronchiolitis was observed more frequently in RV-C cases, although neither difference reached statistical significance. In early studies following its discovery, RV-C was associated with more severe asthma episodes [43,44,45]. A history of asthma as a comorbidity was also associated more frequently with RV-C in the current study. RV-C is more frequently detected at lower ages, with a steady shift towards RV-A later in life, possibly based on a greater immunogenicity of RV-C [46,47]. The observed differences in the clinical presentation could thus be influenced by host factors rather than being based on a species-specific origin. In line with the notion of an underrepresentation of RV-B in non-African studies [33,48], the analysis of the clinical presentation of RV-B was hampered by a low case number. However, RV-B has previously been associated with a decreased clinical severity, as well as a lower replication rate and lower cellular toxicity in vitro [49,50]. In relation to other respiratory pathogens studied at the same hospital, RV showed the highest association with asthma when compared to influenza B virus or parainfluenza virus type 3 infections. The clinical severity was lower with regard to pneumonia and ICU admissions than what was reported for enterovirus B and D and influenza B virus infections [51,52,53].

Increasing evidence suggests that co-infections with other pathogens have to be considered when evaluating the illness severity of rhinovirus infections [33]. An increased length of stay at the hospital, an increased rate of pneumonia, the need for an ICU stay, and assisted ventilation are correlates of the increased severity observed for cases with a co-infection in the current study. Co-infections have been reported in up to 78.9% of the studied cases [41,42]. The frequency of the detected pathogen type thereby seems to undergo a shift from predominantly viral in young children to bacterial with increasing age [23,41,48]. In line with these findings, viral co-infection predominated in the current study population (Table 2). Specific disease entities, however, may still be predominantly associated with bacteria, as reported for CAP and *Streptococcus pneumoniae* co-infections in pneumonia [54]. Nevertheless, the associations with other co-infecting pathogens are not easy to evaluate. While bacterial colonization and a subsequent infection could be enhanced by a rhinovirus infection [2], the interactions with other viral pathogens are more difficult to assess. Modes of such interaction include an upregulation of adhesion molecules [55,56], bacterial internalization [57], disruption of the epithelial barrier [58], and impairment of innate host responses [59] and have been described in vitro for, e.g., *Haemophilus* spp., *Streptococcus pneumoniae*, and *Staphylococcus aureus*. In contrast, in a prospective multicenter study on the cause of CAP in children [60], rhinovirus was the only pathogen that was as frequently detected in healthy controls as in the study cohort. In part, this may be attributed to a long shedding of rhinovirus, even after an acute infection [61,62,63,64,65], with possible coincidental detection of both pathogens. The spectrum of the bacterial pathogens found is in line with data from a recent German pediatric exacerbation cohort [7]. Interestingly, adenovirus was the most frequent viral co-pathogen, followed by bocavirus and paramyxoviruses, such as metapneumovirus and RSV (Table 3). Similar patterns were observed in other studies [41,42,48]. As previously reported for the adult cohort [23], influenza virus was rarely detected as a co-pathogen, which further indicates some kind of viral interference, which was also reported in the wake of the SARS-CoV-2 pandemic (reviewed in [33]). Furthermore, the divergent RV species distribution between cases with co-infections and cases with only RV detection implies that there may be different modes of interaction between RV-A and RV-C and other respiratory pathogens. In part, this may be explained by the usage of different host receptors [2].

Notably, there are several limitations of this study. The genotyping approach was not successful for all detected rhinoviruses and may be a source of bias, although it was also observed in other studies [23,41]. A possible explanation might be the higher sensitivity of the nucleic acid amplification test used for the rhinovirus detection targeting the highly conserved 5’UTR with the amplification of a shorter fragment of 204 bp. Additionally, species-specific differences in the sensitivity of the utilized genotyping protocols cannot be ruled out and are underlined by the better overall performance of the VP4/VP2 protocol for RV-C and the VP1 protocol for RV-A and RV-B. However, the use of two genotyping approaches complementing each other also offered advantages in the detection of 136 distinct genotypes and illustrates the complexity associated with the characterization of a highly diverse virus population. Recombination events, especially in the cases of the five documented RV co-infections of the same species, cannot be ruled out, as a whole genome analysis was not performed. However, due to the rarity of recombination events in rhinoviruses in general, and in the capsid region in particular, co-infections were regarded as more plausible. Furthermore, the diversity of RV species in the 11 cases with RV co-infections resembled the species diversity of the whole study cohort. Asthma was probably underestimated due to the need for lung function tests for an appropriate diagnosis, which cannot be undertaken with patients under the age of five. Due to the retrospective study design, only associations could be shown, without proof of causality. Patient selection favoring severe cases may have occurred due to the sampling at a tertiary care hospital, which is underlined by the high numbers of pneumonia cases and cases that needed an ICU stay. Detection of a further pathogen was considered as a co-infection; however, especially for bacteria, colonization cannot be ruled out. Finally, due to the limited number of RV-B cases, the study lacks the power to determine potential further species-specific differences in comparison to RV-A and RV-C.

## 5. Conclusions

This study reports on the epidemiology and associated clinical spectrum of RVs in pediatric patients who were treated at a tertiary care university hospital in Germany. The present report on infection patterns is consistent with other studies and indicates a differential circulation between adult and pediatric patients at the same time. Furthermore, the tremendous genetic diversity and the adjunct complexity of rhinoviruses’ epidemiology are highlighted. Larger population-based surveillance programs are needed to determine shifts in predominating RV genotypes and for the assessment of possible genotype-specific disease associations. The indication of divergent clinical presentations may necessitate a greater focus on RV species differentiation to facilitate further research. Additionally, the importance of co-infections for the assessment of RV-associated disease severity is underlined. To overcome the high economic burden of RV-associated illness, the implementation of new treatment and vaccination strategies [66,67] with a focus on RV-A and RV-C is warranted. 

## Figures and Tables

**Table 1:** Gender, age, and rhinovirus species distribution during the study period.

		Season 2013/2014	Season 2014/2015	Season 2015/2016	Season 2016/2017	Total		*p*-Value
age (years)	[mean ± SD]	1.99 ± 2.68	2.58 ± 3.31	2.07 ± 3.15	2.8 ± 3.9	2.42 ± 3.37		n.s.
male	[% (n/total)]	61.5 (91/148)	60.3 (132/219)	58.3 (105/180)	55.5 (127/229)	58.6 (455/776)	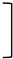	n.s.
female	[% (n/total)]	38.5 (57/148)	39.7 (87/219)	41.7 (75/180)	44.5 (102/229)	41.4 (321/776)		
RV species								
RV-A	[% (n/total)]	51.4 (76/148)	49.3 (108/220)	49.4 (89/180)	47.2 (108/229)	49.1 (381/776)	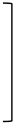	
RV-B	[% (n/total)]	6.1 (9/148)	5.5 (12/220)	6.1 (11/180)	6.1 (14/229)	5.9 (46/776)		n.s.
RV-C	[% (n/total)]	39.9 (59/148)	44.7 (98/220)	41.7 (75/180)	46.3 (106/229)	43.6 (338/776)		
multiple	[% (n/total)]	2.7 (4/148)	0.5 (1/220)	2.8 (5/180)	0.4 (1/229)	1.4 (11/776)		

Analyzed categories are displayed in the column to the left and either given as relative and absolute frequencies [% (n/total)] or ranges [median ± SD]. (n/total) indicates the respective cases among the total amount of cases for the respective season. n.s., not significant. The Kruskal–Wallis test was used to analyze the age.

**Table 2:** Study population and clinical features of RV cases.

		RV-A 49.8% (381/765)	RV-B 6% (46/765)	RV-C 44.2% (338/765)	Total		*p*-Value
**study population**							
female	[% (n/total)]	39.4 (150/381)	43.5 (20/46)	42.3 (143/338)	40.9 (313/765)	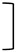	n.s.
male	[% (n/total)]	60.6 (231/381)	56.5 (26/46)	57.7 (195/338)	59.1 (452/765)		
age (years)	[mean ± SD]	2.62 ± 3,57	3.5 ± 5.17	2.07 ± 2.77	2.43 ± 3.38		n.s.
inpatients	[% (n/total)]	97.6 (372/381)	95.7 (44/46)	98.8 (334/338)	98 (750/765)	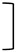	n.s.
outpatients	[% (n/total)]	2.4 (9/381)	4.3 (2/46)	1.2 (4/338)	2 (15/765)		
length of stay (days)	[median(range)]	5 (1–85)	6 (1–77)	5 (1–87)	5 (1–87)		0.007
**comorbidities and risk factors**							
asthma	[% (n/total)]	7.4 (28/377)	4.3 (2/46)	14.3 (48/336)	10.3 (78/759)		0.004
COPD	[% (n/total)]	0.5 (2/377)	0 (0/46)	0 (0/337)	0.3 (2/760)		n.s.
structural lung disease	[% (n/total)]	7.4 (28/377)	15.2 (7/46)	5.3 (18/337)	7.0 (53/760)		n.s.
airway ass. allergy	[% (n/total)]	9.1 (34/374)	2.2 (1/45)	10.1 (34/335)	9.2 (69/754)		n.s.
cardiovascular diseases	[% (n/total)]	13.0 (49/328)	2.2 (1/46)	5.9 (20/337)	9.2 (70/760)		0.001
metabolic disease	[% (n/total)]	4.0 (15/377)	8.7 (4/46)	3.3 (11/337)	3.9 (30/760)		n.s.
malignancy	[% (n/total)]	7.7 (29/378)	10.9 (5/46)	3.6 (12/325)	6.0 (46/761)		n.s.
immunosuppression	[% (n/total)]	8.6 (32/373)	11.4 (5/44)	6.5 (22/337)	7.8 (59/754)		n.s.
**clinical presentation**							
symptomatic	[% (n/total)]	95.5 (360/377)	89.1 (41/46)	96.7 (327/338)	95.7 (728/761)		n.s.
temperature							
<37.5 °C	[% (n/total)]	42.7 (159/372)	39.5 (17/43)	53.9 (181/336)	47.5 (357/751)	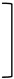	
37.5–37.9 °C	[% (n/total)]	10.2 (38/372)	9.3 (4/43)	13.7 (46/336)	11.7 (88/751)		0.001
≥38 °C	[% (n/total)]	47.0 (175/372)	51.2 (22/43)	32.4 (109/336)	40.7 (306/751)		
dyspnea	[% (n/total)]	54.4 (203/373)	43.2 (19/44)	65.7 (222/338)	58.8 (444/755)		<0.001
bronchodilator usage	[% (n/total)]	61.5 (230/374)	37.2 (16/43)	73.6 (248/337)	65.5 (494/754)		<0.001
URTI	[% (n/total)]	83.8 (315/376)	75.6 (34/45)	84.9 (286/337)	83.8 (635/758)		n.s.
pharyngitis	[% (n/total)]	75.7 (284/375)	68.9 (31/45)	74.2 (250/337)	74.6 (565/757)		n.s.
rhinitis	[% (n/total)]	55.2 (207/375)	40.0 (18/27)	56.1 (189/337)	54.7 (414/757)		n.s.
tonsillitis	[% (n/total)]	22.9 (86/375)	22.2 (10/45)	22.6 (76/337)	22.7 (172/757)		n.s.
LRTI	[% (n/total)]	57.7 (217/376)	44.4 (20/45)	64.5 (218/338)	59.9 (455/759)		n.s.
pneumonia	[% (n/total)]	17.3 (65/375)	13.3 (6/45)	8.3 (28/310)	13.1 (99/758)		0.002
bronchiolitis	[% (n/total)]	43.1 (162/376)	35.6 (16/45)	57.4 (19/338)	49.0 (372/759)		<0.001
ICU stay	[% (n/total)]	23.6 (89/377)	28.3 (13/46)	20.4 (69/338)	22.5 (171/761)		n.s.
ventilation							
none	[% (n/total)]	83.1 (309/372)	86.4 (38/44)	86.9 (291/335)	85.0 (638/751)	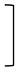	
non-invasive	[% (n/total)]	8.1 (31/372)	4.5 (2/44)	7.8 (26/335)	7.9 (59/751)		n.s.
invasive	[% (n/total)]	8.4 (32/372)	9.1 (4/44)	5.4 (18/335)	7.2 (54/751)		
co-infection	[% (n/total)]	42.0 (160/381)	34.8 (16/46)	27.8 (94/338)	35.3 (270/765)		<0.001
bacterial	[% (n/total)]	16.3 (62/381)	15.2 (7/46)	8.9 (30/338)	12.9 (99/765)	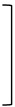	
viral	[% (n/total)]	20.7 (79/381)	17.4 (8/46)	16.6 (56/338)	18.7 (143/765)		n.s.
fungal	[% (n/total)]	0.3 (1/381)	0 (0/46)	0 (0/338)	0.1 (1/765)		
combined	[% (n/total)]	4.7 (18/381)	2.2 (1/46)	2.4 (8/338)	3.5 (27/765)		

Analyzed categories are displayed in the column to the left and are either given as frequencies (%), range [median(range)], or as means and standard deviations (mean ± SD). (n/total) indicates the respective cases for the total amount of available data. The *p*-values of the Kruskal–Wallis test and of the Chi-square tests for the contingency tables including all three RV species are indicated. All significant *p*-values for the pairwise analysis of interspecies differences are given in the corresponding paragraph. COPD, chronic obstructive pulmonary disease; ass., associated; URTI, upper respiratory tract infection; LRTI, lower respiratory tract infection; ICU, intensive care unit; n.s., not significant. The Kruskal–Wallis test was used to analyze the age and length of stay.

**Table 3:** Distribution of co-infecting pathogens.

Bacteria	n	Viruses	n	Fungi	n
*Haemophilus influenzae*	26	Adenovirus	61	*Aspergillus* spp.	2
*Staphylococcus aureus*	19	Bocavirus	44	*Candida* spp.	1
*Streptococcus pneumoniae*	17	RSV	44		
*Escherichia coli*	15	Metapneumovirus	15		
*Moraxella catarrhalis*	15	Coronavirus OC43	9		
*Klebsiella pneumoniae*	14	Enterovirus	8		
*Pseudomonas aeruginosa*	11	Parainfluenzavirus type 3	5		
*Enterobacter cloacae*	7	Parainfluenzavirus type 4	5		
*Klebsiella oxytoca*	7	Coronavirus NL63	4		
*Mycoplasma pneumoniae*	7	Parainfluenzavirus type 1	4		
*Chlamydophila pneumoniae*	6	Parainfluenzavirus type 2	4		
*Haemophilus parainfluenzae*	5	Coronavirus HKU1	3		
*Streptococcus pyogenes*	5	CMV	2		
*Serratia marcescens*	4	Influenza A H1N1	2		
*Stenotrophomonas maltophilia*	4	Influenza A H3N2	2		
*Acinetobacter baumanii*	2	EBV	1		
*Morganella morganii*	2	HHV 6	1		
*Bordetella pertussis*	1	HSV 1	1		
*Citrobacter freundii*	1				
*Enterobacter aerogenes*	1				
*Enterobacter hormaechei*	1				
*Enterobacter kobei*	1				
*Haemophilus haemolyticus*	1				
*Haemophilus parahaemolyticus*	1				
*Leclercia adecarboxylata*	1				
*Pantoea agglomerans*	1				
*Proteus mirabilis*	1				
*Pseudomonas putida*	1				

Pathogens detected by type, with n showing their frequencies of detection.

**Table 4:** Comparison of samples with and without co-infections.

		RV Only 64.7 (495/765)	RV + Co-Infection 35.3 (270/765)	Total	*p*-Value	
**season**						
season 2013/2014	[% (n/total)]	17.4 (86/495)	21.5 (58/270)	18.8 (144/765)	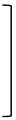	n.s.
season 2014/2015	[% (n/total)]	29.7 (147/495)	26.3 (71/270)	28.5 (218/765)		
season 2015/2016	[% (n/total)]	21.0 (104/495)	26.3 (71/270)	22.9 (175/765)		
season 2016/2017	[% (n/total)]	31.9 (158/49)	25.9 (70/270)	29.8 (228/765)		
**study population**						
female	[% (n/total)]	42.2 (209/495)	38.5 (104/270)	40.9 (313/765)	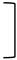	n.s.
male	[% (n/total)]	57.8 (286/495)	61.5 (166/270)	59.1 (452/765)		
length of stay (days)	[median (range)]	4 (1–87)	6 (1–82)	5 (1–87)	<0.001	
**RV species**						
RV-A	[% (n/total)]	44.6 (221/495)	59.3 (160/270)	49.8 (381/765)	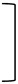	
RV-B	[% (n/total)]	6.1 (30/495)	5.9 (16/270)	6.0 (46/765)		<0.001
RV-C	[% (n/total)]	49.3 (244/495)	34.8 (94/270)	44.2 (338/765)		
**clinical presentation**						
symptomatic	[% (n/total)]	93.9 (461/491)	98.9 (267/270)	95.7 (728/761)	0.001	
temperature						
<37.5 °C	[% (n/total)]	51.1 (246/481)	41.1(111/270)	47.5 (357/751)	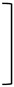	
37.5–37.9°C	[% (n/total)]	11.4 (55/481)	12.2 (33/270)	11.7 (88/751)		0.025
≥38 °C	[% (n/total)]	37.4 (180/481)	46.7 (126/270)	40.7 (306/751)		
dyspnea	[% (*n*/total)]	59.2 (287/485)	58.1 (157/270)	58.8 (444/755)	n.s.	
bronchodilator usage	[% (n/total)]	65.1 (315/484)	66.3 (179/270)	65.5 (494/754)	n.s	
URTI	[% (*n*/total)]	82.2 (401/488)	86.7 (234/270)	83.8 (635/758)	n.s.	
pharyngitis	[% (*n*/total)]	71.9 (350/487)	79.6 (215/270)	74.6 (565/757)	0.019	
rhinitis	[% (*n*/total)]	54.0 (263/487)	55.9 (151/270)	54.7 (414/757)	n.s.	
tonsillitis	[% (*n*/total)]	19.9 (97/487)	27.8 (75/270)	22.7 (172/757)	0.013	
LRTI	[% (*n*/total)]	56.6 (277/489)	65.9 (178/270)	59.9 (455/759)	0.012	
pneumonia	[% (*n*/total)]	7.8 (38/488)	22.6 (61/270)	13.1 (99/758)	<0.001	
bronchiolitis	[% (*n*/total)]	51.1 (250/489)	45.2 (122/270)	49.0 (372/759)	n.s.	
ICU stay	[% (*n*/total)]	17.7 (87/491)	31.1 (84/270)	22.5 (171/761)	<0.001	
ventilation						
none	[% (*n*/total)]	90.4 (435/481)	75.3 (203/270)	85.0 (638/751)	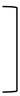	
non-invasive	[% (*n*/total)]	6.2 (30/481)	10.7 (29/270)	7.9 (59/751)		<0.001
invasive	[% (*n*/total)]	3.3 (16/481)	14.1 (38/270)	7.2 (54/751)		

Analyzed categories are displayed in the column to the left and either given as frequencies (%) or medians and ranges [median (range)]. (n/total) indicates the respective cases for the total amount of available data. The *p*-values of the Mann–Whitney U test and of the Chi-square tests for the contingency tables including all subgroups are indicated. The *p*-values for the pairwise analysis for analysis of subgroup differences are given in the corresponding paragraph. A comparison between RV cases with (RV + co-infection) and without (RV only) a documented co-infection with a viral, bacterial, or fungal pathogen was performed. N.s., not significant. The Mann–Whitney U test was used to analyze the length of stay.

## Data Availability

Identified sequences were submitted to GenBank (accession no. ON767112 to ON768616).

## Supplementary Materials

The following supporting information can be downloaded at: https://www.mdpi.com/article/10.3390/v14081829/s1, Figure S1: Combined monthly total numbers of tested samples and rhinovirus cases, Table S1: Primers and probes used for rhinovirus detection, Table S2: Reagent concentration for rhinovirus detection, Table S3: Cycling conditions for rhinovirus detection.
