# Supplementary material for: Human Rhinoviruses in Pediatric Patients in a Tertiary Care Hospital in Germany: Molecular Epidemiology and Clinical Significance
Source: Viruses. 2022 Aug 20;14(8):1829. doi: 10.3390/v14081829 (PMC9415293; doi:10.3390/v14081829)
Supplement: Supplementary file 1 [file viruses-14-01829-s001.zip › viruses-1864276-supplementary.pdf]

Human Rhinoviruses in Pediatric Patients in a Tertiary Care Hospital in Germany: Molecular Epidemiology and Clinical Significance

Franziska Neugebauer, Sandra Bergs, Uwe Gerd Liebert and Mario Hönemann

Supplementary Material

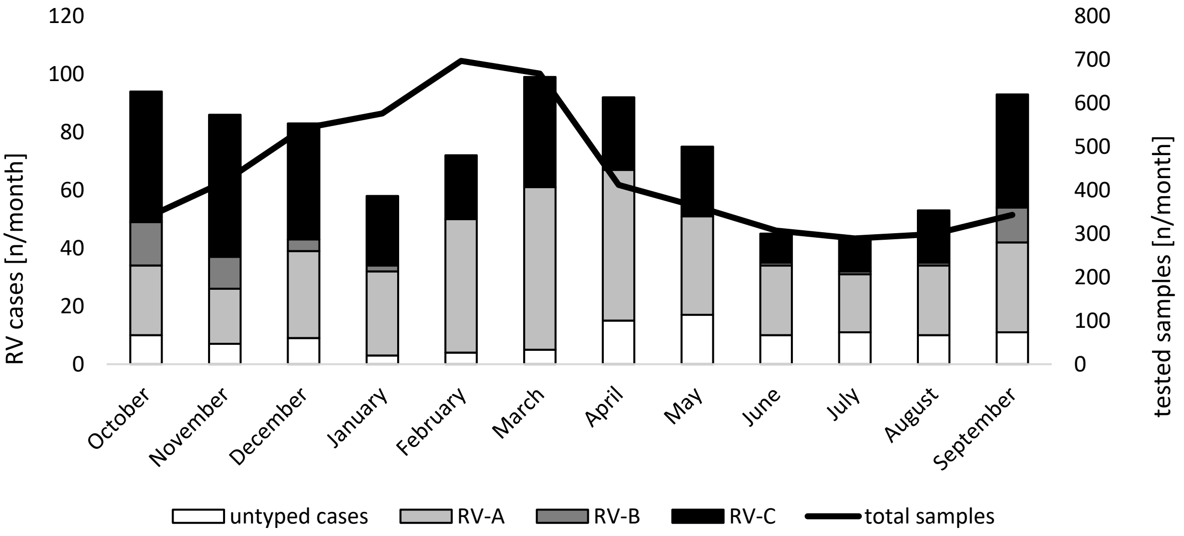

Figure S1: Combined Monthly total numbers of tested samples and rhinovirus cases. Combined Monthly total numbers of tested samples and rhinovirus cases (n = 776) from 2013 to 2017 stratified by species A, B, and C, as well as untypable rhinoviruses. Note the two different y-axes: the left axis shows the absolute numbers of detected Rhinovirus A, Rhinovirus B, and Rhinovirus C cases, as well as the absolute numbers of untyped cases, while the right axis shows the absolute number of tested samples.

Table S1. Primers and Probes used for Rhinovirus detection.

**Primer/Probe**	**Sequence**
Primer HRV-LNA-for (forward, 14 nt)	5′- CY+ AGC C+T GCG TGG C –3
Primer HRV-rev (reverse, 21 nt)	5′- GAA ACA CGG ACA CCC AAA GTA –3
Taqman-Probe HRV-Taq-FAM (22 nt)	5′- 6FAM- TCC TCC GGC CCC TGA ATG YGG C-BHQ1 -3'

FAM= FL= Fluorescein; BHQ= Black Hole Quencher=Dark Quencher, primer concentration: 10 pmol/µl, probe concentration: 4 pmol/µl.

Table S2. Reagent concentration for Rhinovirus detection.

Reagent	Concentration	Volume	Final Concentration	Company
H_2O		1,7 µl		QIAGEN
2x Fast MP RT-PCR MM	2x(+Mg Cl_2)	10,0 µl	1x	QIAGEN
HRV-LNA-for	10 pmol/µl	0,8 µl	0,4 µM	Metabion
HRV-rev	10 pmol/µl	0,8 µl	0,4 µM	Metabion
HRV-Taq-FAM	4 pmol/µl	1,0 µl	0,2 µM	TibMolBiol
Fast RT Mix		0,2 µl		QIAGEN
RNase Inhibitor	40 Units/µl	0,5 µl	20 Units	Thermo Scientific
RNA		5,0 µl		

Reagent concentrations and volumes used for Rhinovirus detection.

Table S3. Cycling conditions for Rhinovirus detection.

Step	Temperature	Duration	Ramp Rate	Mode	
RT	50°C	20:00 min.	20.00°C/s	None	
Denaturation/Activation	95°C	5:00 min.	20.00°C/s	None	
Amplification	95°C	0:15 min.	20.00°C/s	None	45 Cycles
	56°C	0:10 min.	20.00°C/s	None	
	60°C	0:30 min.	20.00°C/s	Single(530) nm)	
Cooling	40°C	0:30 min.	20.00°C/s	None	

The amplification was performed on a Light Cycler 2.0 Instrument (Roche).
